# Household migration and children’s diet in Nepal: an exploratory study

**DOI:** 10.1186/s13104-019-4430-x

**Published:** 2019-07-11

**Authors:** Yubraj Acharya, Dirgha J. Ghimire, Prem Bhandari, Ramesh Ghimire, Andrew D. Jones

**Affiliations:** 1 Department of Health Policy and Administration, The Pennsylvania State University, 601L Ford Building, University Park, PA 16802 USA; 2 The Institute for Social and Environmental Research, Chitwan, Nepal; 3 The Institute for Social Research, University of Michigan, Ann Arbor, USA; 4 Department of Development Studies, Kathmandu University, Kathmandu, Nepal; 5 Department of Nutritional Sciences, University of Michigan, School of Public Health, Ann Arbor, USA

**Keywords:** Nepal, Migration, Dietary diversity

## Abstract

**Objective:**

Individuals from low-income countries often migrate abroad for employment. The association between such migration and investment in education as well as other societal and familial outcomes has previously been examined. However, we have a limited understanding of the association between migration and children’s nutrition. We aim to determine the extent to which migration of household members influences children’s diet in a semi-urban region of Nepal.

**Results:**

In our study setting, children in households with a migrant had higher dietary diversity scores, 0.69 on average, than their counterparts in households without a migrant. These children were approximately 43% points more likely to meet a minimum requirement for dietary diversity. These differences originated primarily from higher consumption of meat (41% points) and eggs (20% points). Approximately 37 percent of children in the sample consumed processed food during the 24 h preceding the survey. However, we found no evidence that migration was associated with the consumption of processed foods or with reduced frequency of breastfeeding. Our finding that migration is associated with higher consumption of meat and eggs is particularly encouraging, given that the protein deficiency in Nepal is estimated to be nearly 43 percent.

**Electronic supplementary material:**

The online version of this article (10.1186/s13104-019-4430-x) contains supplementary material, which is available to authorized users.

## Introduction

In recent years, the potential for agriculture to influence nutrition of infants and children in rural, low-income circumstances has received much attention [1]. The linkages between agriculture and nutrition have been broadly examined [2], though most research has focused on the impact of increasing production or income from agriculture on household consumption. While these are undoubtedly important pathways, structural transformations in rural regions of low-income countries are driving farming families to earn increasingly large proportions of their income from non-agricultural sources, often from migrant labor opportunities within their home countries or abroad [3]. In fact, global population mobility is one of the most striking demographic changes witnessed during the twenty-first century [4].

There are several mechanisms by which migration may influence the agriculture-nutrition nexus. Remittances from migrant household members, for example, may increase household incomes allowing for the purchase of land, animals, seed, and fertilizer that could enhance agricultural production, and potentially nutrition through improved household food security [4, 5]. Additional income may also be used to purchase more food, higher quality food, or health care inputs that could generate direct nutritional benefits. Access to new educational opportunities through migration may also make individuals more aware of the critical role that nutrition plays in the growth and development of children. Conversely, however, migrants’ departure from the household increases the work burden of non-migrant household members, especially women [6, 7]. Additional agricultural labor responsibilities for women left behind could detract from breastfeeding and other caregiving responsibilities that are critical for the healthy growth and development of children [8, 9]. At the same time, though migration may afford access to new kinds of healthy foods, it may also provide new access to highly processed foods that could adversely impact nutrition and health outcomes. Migration then, is instrumental in understanding the changing livelihoods of households as well as how these changes may influence nutrition outcomes. Yet, remarkably little research has examined these relationships [10].

## Main text

### Methods

#### Setting

We conducted this study in the Chitwan Valley of southern Nepal. In 2011, approximately, 8.3% of the population lived abroad [11]. Remittances from migrant workers abroad currently constitute about 29% of Gross Domestic Product (GDP) [12], and close to 50% of Nepalis rely on financial help from relatives abroad, among the highest rates in the region [11]. The country also faces a substantial burden of both undernutrition (i.e. more than two-fifths of under-five children are growth stunted, and more than one-third of women are anemic [13]), as well as an increasing burden of overweight [13]).

#### Study design

We randomly selected 250 households from 40 neighborhoods in a larger longitudinal study called the Chitwan Valley Family Study (CVFS) using a simple random sampling technique. Trained enumerators visited the households between May and August 2013 and collected basic demographic information. In 51 households which had at least one child below the age of 2 years, we administered a quantitative 24-h dietary recall instrument using standard protocols [14] to assess the dietary intake of children in the 24 h preceding the interview.

#### Outcome variables

Our primary outcome was the diversity of young children’s diets. We created diet diversity scores for each child aged 6–24 months by summing the number of food groups consumed by the child in the 24 h prior to the interview as reported by a primary caregiver. We calculated seven food groups based on those used to calculate the World Health Organization’s Minimum Dietary Diversity (MDD) indicator for young children—a metric that has been validated as an indicator of diet quality based on the micronutrient adequacy of children’s diets [15]. The seven food groups included: (1) grains, roots and tubers; (2) legumes and nuts; (3) dairy products (including milk, yogurt and cheese); (4) flesh foods (including meat, fish, poultry, and organ meats); (5) eggs; (6) vitamin A-rich fruits and vegetables; and (7) other fruits and vegetables.

We further calculated the MDD indicator as a binary variable indicating whether a child consumed at least four of the seven food groups in the previous 24 h. In addition, we separately examined binary variables representing consumption of four of the seven food groups that compose the diet diversity score. Each of the seven food groups make an important contribution to the overall micronutrient adequacy of children’s diets [16]. However, all children in the sample were given three of the seven food components during the 24 h preceding the survey. Therefore, we evaluated the association between migration and the remaining four food groups: (1) legumes and nuts; (2) dairy products; (3) flesh foods; and (4) eggs. We also used the consumption of processed foods (e.g., noodles, biscuits, and beaten rice) available in the market as an outcome. Finally, we examined the number of times the child was breastfed during the previous 24 h.

#### Explanatory variables

The main explanatory variable was whether the household had at least one member away from the household for 6 months or more at any point in the past. We also measured several factors that we hypothesized could influence an individual’s decision to migrate, as well as those factors that may affect a child’s diet. These included child’s gender and age, mother’s education, household’s annual income from sources other than remittances, the amount of land available for agriculture, the number of poultry owned and livestock owned, the number of household members, and the household’s ethnicity.

#### Estimation

In order to assess the relationship between a household’s migration status and the various nutritional outcomes and intermediate factors, we estimated the following equation:

1 $$Y_{ij} = \alpha + \beta_{1} \,Migrant_{j} + \varvec{X}_{ij} + \varepsilon$$

In Eq. (1), Y_*ij*_ is the nutritional outcome of interest for a child *i* in household *j*. *Migrant* is a binary variable which equals one if a member has been away from home for at least six months in the past, so varies by household. **X** represents those child-, maternal- and household-level characteristics described above.

The coefficient β_1_ reflects the association between migration and the outcome. For example, β1 > 0 would be indicative of migration’s positive association with diet.

We used an ordinary least square (OLS) regression method for all outcomes. In the regression with the dietary diversity index as the outcome, the coefficient should be interpreted as the additional number of dietary factors associated with migration. For example, if β1 = 1, it implies that children in households with a migrant consume one additional diet component relative to children in households without a migrant. For the binary outcomes, we can interpret the coefficient multiplied by 100 as the percentage change in the outcome associated with migration. For example, if β1 = 0.41 in a regression with meat consumption as the outcome, it means that children in households with a migrant are 41% points (= 0.41 × 100) more likely to have consumed meat during the 24 h preceding the survey relative to the children in households without a migrant.

We report bootstrapped standard errors, with observations randomly selected from the sample with replacement (wild bootstrap), to alleviate concerns about inaccurate standard errors resulting from the small sample.

### Results

#### Descriptive statistics

Of the 205 households, 51 had children between the age of 6 months and 2 years. They form the analytic sample. Descriptive statistics of the primary outcome and main explanatory variables, as well as several covariates used in the analysis, are shown in Table 1.

**Table 1:** Summary statistics for the overall sample (n = 51)

	N (%), or mean	Sd
Child’s age, months	15.73	5.33
Child is female	26 (51%)	
Mother’s education, years	9.88	3.55
Number of household members	5.73	2.42
Farming is a source of livelihood	35 (68.3%)	
Amount of land being farmed, *kattha*	9.65	10.92
Number of chickens and ducks	5.43	7.86
Number of livestock	2.41	2.68
Annual household income, %		
Below Rs. 25,000	1 (1.96%)	
Between Rs 25,000–50,000	7 (13.73%)	
Between Rs. 50,000–100,000	18 (35.29%)	
Between Rs. 100,000–250,000	14 (27.45%)	
Between Rs. 250,000–500,000	7 (13.73%)	
More than Rs. 500,000	4 (7.84%)	
Caste/ethnic group, %		
Brahman, Chhetri	15 (29.41%)	
Newar	13 (25.49%)	
*Janajati*	10 (19.61%)	
*Hill Dalits*	1 (1.96%)	
*Tarai Dalits*	12 (23.53%)	
Diet diversity score	4.19	1.09
Diet diversity score at least 4	33 (64.7%)	
In the previous 24 h, the child was given…		
Grains	51 (100%)	
Lentils	18 (35.29%)	
Dairy	17 (33.33%)	
Flesh	19 (37.25%)	
Eggs	7 (13.73%)	
Vitamin A vegetables	51 (100%)	
Fruits	51 (100%)	
Processed food	19 (37.25%)	
Number of times the child was breastfed in the previous 24 h	8.60	3.11

This table represents the descriptive statistics for the 51 children included in the analysis. All variables were self-reported by the respondents

The average dietary diversity score was 4.2 (sd = 1.1). Sixty-four percent of children met the MDD indicator. During the 24 h prior to the survey, all children were given three of the seven diet components: (1) grains, (2) vitamin A-rich fruits and vegetables, and (3) other fruits. Given the absence of any variation in these components, we did not evaluate the association between these components and migration. In the sample, consistent with the protein deficiency among children in Nepal [17], approximately one-third of the children were fed lentils, dairy, or meat in the previous 24 h (35%, 33% and 37%, respectively). Thirteen percent of children were given eggs. Thirty-seven percent of children in the sample were fed processed food available in the market. On average, a child was breastfed 8.6 times during the previous 24 h.

#### Multivariate results

The coefficients and the standard errors from estimating the regression in Eq. (1) are in Table 2 (see Additional file 1: Table S2 for results from step-wise regressions). The unadjusted values in column (1) show that, on average, children in non-migrant households had a dietary diversity index of 3.75 (CI 3.33, 4.17). The index for children in migrant households was 0.73 points higher (CI 0.15, 1.31); in other words, on average, children in migrant households consumed three-fourths of an item more than those in non-migrant households. After we adjusted for various characteristics of the child, the mother and the household, the difference fell to 0.69 (CI 0.05, 1.33) (column 2).

**Table 2:** Results from the regression of diet outcomes on migration status

	Diet diversity score (continuous)		Minimum diet diversity (binary)	
	Unadjusted	Adjusted	Unadjusted	Adjusted
Migrant household	0.734**	0.693**	0.324**	0.428***
	(0.293)	(0.327)	(0.138)	(0.145)
Household income		− 0.022		− 0.058
		(0.157)		(0.056)
Amount of agricultural land		0.007		0.004
		(0.027)		(0.010)
Total number of poultry		0.019		0.011
		(0.023)		(0.009)
Total number of livestock		− 0.058		− 0.074*
		(0.104)		(0.042)
Household's ethnic category		− 0.113		− 0.010
		(0.118)		(0.056)
Mother's education		0.072		0.026
		(0.048)		(0.023)
Household size		− 0.12		− 0.045
		(0.088)		(0.036)
Age of the child		0.009		0.009
		(0.027)		(0.013)
Gender of the first respondent		0.079		0.087
		(0.337)		(0.148)
Constant	3.750***	3.933***	0.450***	0.567
	(0.214)	(1.059)	(0.113)	(0.401)
N	51	51	51	51
R-squared	0.11	0.30	0.11	0.41
Adjusted R-squared	0.09	0.13	0.09	0.26

The table shows coefficients from estimating Eq. (1) using a linear regression model on the sample whose descriptive statistics are in Table 1. Standard errors are in parenthesis. *p < 0.10, **p < 0.05, ***p < 0.01. In the adjusted models, household income and ethnic category are included as continuous values. The results do not change significantly when household income and ethnic category are included as categorical variables (see Additional file 1: Table S1) In the first two columns, the coefficient on ‘migrant household’ should be read as the amount of increase in a child’s diet diversity resulting from his or her household member’s migration. In the remaining two columns, the coefficient multiplied by 100 gives the percentage point change in the probability that the minimum diet diversity is met, again due to a household member’s migration

Unadjusted values from the regression of MDD as the outcome (column 3) showed that, on average 45% of children in non-migrant households met the MDD indicator. In households with a migrant, an additional 32% of children met the MDD requirement; for these children, the probability of meeting the MDD indicator of four food groups was approximately 77%. The difference in the probability of meeting the MDD indicator between children in migrant vs. non-migrant households was larger—42% points—after we controlled for household characteristics (column 4).

The differences in the dietary diversity index and the MDD indicator between migrant and non-migrant households were driven by the higher consumption of meat and fish, and eggs. For these components, the coefficients on the household’s migration status were statistically significant at the five percent level (Table 3). In other components of diet, the coefficients were all statistically insignificant.

**Table 3:** OLS results from the regression of diet components on migration status

	Lentils	Dairy	Flesh	Egg	Processed food	Times breastfed
Overall mean	0.35	0.33	0.37	0.13	0.37	8.6
Migrant household	0.017	0.073	0.406**	0.196**	0.115	− 0.558
	(0.146)	(0.153)	(0.166)	(0.088)	(0.168)	(1.089)
Household income	− 0.083	0.002	0.038	0.021	− 0.042	− 0.06
	0.062)	(0.062)	(0.069)	(0.058)	(0.062)	(0.460)
Amount of agricultural land	0.003	− 0.001	− 0.001	0.006	0.014	− 0.01
	0.010)	(0.011)	(0.009)	(0.009)	(0.012)	(0.076)
Total number of poultry	− 0.001	0.006	0.003	0.011	0.004	− 0.028
	(0.010)	(0.009)	(0.010)	(0.009)	(0.011)	(0.056)
Total number of livestock	− 0.007	0.028	− 0.070*	− 0.010	− 0.041	0.200
	(0.043)	(0.043)	(0.036)	(0.028)	(0.037)	(0.341)
Household’s ethnic category	− 0.056	− 0.109**	0.073	− 0.020	− 0.020	0.077
	(0.054)	(0.049)	(0.060)	(0.030)	(0.064)	(0.345)
Mother’s education	0.051**	0.029	− 0.007	− 0.001	− 0.006	0.006
	(0.024)	(0.023)	(0.023)	(0.012)	(0.025)	(0.166)
Household size	− 0.044	− 0.043	− 0.032	− 0.001	− 0.040	− 0.418
	(0.039)	(0.037)	(0.039)	(0.027)	(0.037)	(0.311)
Age of the child	0.001	0.0000	0.009	− 0.001	0.033**	− 0.208**
	(0.014)	(0.013)	(0.014)	(0.010)	(0.015)	(0.096)
Gender of the first respondent	0.093	0.185	− 0.012	− 0.186*	− 0.090	0.715
	(0.163)	(0.140)	(0.143)	(0.106)	(0.160)	(0.800)
Constant	0.552	0.344	0.03	0.008	0.296	14.030***
	(0.485)	(0.461)	(0.441)	(0.341)	(0.575)	(3.138)
N	51	51	51	51	51	50
R-squared	0.28	0.29	0.29	0.31	0.21	0.21
Adjusted R-squared	0.10	0.11	0.11	0.14	0.01	0.01

The table shows results from estimating Eq. (1) on the sample whose descriptive statistics are in Table 1. Each column represents a separate regression. Standard errors are in parenthesis. *p < 0.10, **p < 0.05, ***p < 0.01. In the adjusted models, household income and ethnic category are included as continuous values. The coefficient on ‘migrant household’ multiplied by 100 gives the percentage point change in the probability that the specific food component is given to the child during the 24 h preceding the survey

We found no evidence that migration status was associated with the consumption of processed food items available from the market (Table 3, column 6). We also found no evidence that children in migrant households were breastfed fewer times during the previous 24 h than children in non-migrant households (Table 3, column 7).

### Discussion

In this setting in Nepal, temporary migration of household members was associated with more diverse diets among children. Given the high prevalence of protein deficiency in the country, it is encouraging that migration was associated with higher consumption of flesh foods and eggs in particular.

In terms of the mechanisms, it is difficult to attribute the observed associations in our study to a specific factor. Based on prior evidence from other settings, three mechanisms may be especially salient. First, international exposure of the migrant may increase a household’s knowledge of diet and other health-improving practices, such as using clean water [18, 19], through exposure to a wider range of experiences [20]. This argument is consistent with our findings because we do not see any association of migration with the child’s intake of processed food from the market which tend to be less nutritious.

Second, migration of male household members may improve the autonomy of women left behind who may be more careful about investing in children’s health. A number of previous studies have found that women are more likely than men to prioritize the basic food and health care needs of their children over other needs [21–23]. Finally, cash remittances may allow the food purchaser, who is often the woman in this setting, to purchase higher quality food.

That 37% of the children in the sample were fed processed food from the market is a major policy concern, even though migration does not seem to be driving this behavior. The children in the sample are within the “golden 1000 days”—the most critical period for a child’s cognitive and physical development, as evidenced by the medical literature [24]. The consumption of such food by older children is likely even higher and may help explain the rising incidence of child obesity in the country.

Studies with larger samples and appropriate measures will be required to disentangle these mechanisms as well as to provide more concrete policy options on how Nepal and countries like it that are undergoing rapid migration, can use this dynamic to their long-term advantage to improve investments in children’s health.

## Limitations

1. Given the cross-sectional nature of our data, we cannot interpret the observed associations as causal.
2. The findings may have limited external validity, and regression models are vulnerable to overfitting, given the small sample.

## Additional file

**Additional file 1.** Additional tables.

## Data Availability

The datasets used and/or analyzed during the current study are available from the corresponding author on reasonable request.

## Abbreviations

CVFS: Chitwan Valley Family Study
GDP: Gross Domestic Product
MDD: Minimum Dietary Diversity
OLS: ordinary least squares
