# Supplementary material for: Household migration and children’s diet in Nepal: an exploratory study
Source: BMC Res Notes. 2019 Jul 11;12:390. doi: 10.1186/s13104-019-4430-x (PMC6625056; doi:10.1186/s13104-019-4430-x)
Supplement: Supplementary file 1 — Additional file 1. Additional tables. [file 13104_2019_4430_MOESM1_ESM.docx]

**Household Migration and Children's Diet: An Exploratory Study**
Additional Tables

**Additional Table S1**. Results from the Regression of Diet Outcomes on Migration Status with Income and Caste/Ethnicity Included as Categorical Variables

|  |  | Diet Diversity Score | Minimum Diet Diversity |
| --- | --- | --- | --- |
|  |  |  |  |
| Migrant household |  | 0.752** | 0.416*** |
|  |  | (0.355) | (0.146) |
| Household income (ref: below Rs. 25,000) | | |  |
| Between Rs 25,000-50,000 |  | -0.382 | -0.186 |
|  |  | (1.171) | (0.483) |
| Between Rs. 50,000-100,000 |  | -0.864 | -0.168 |
|  |  | (1.126) | (0.464) |
| Between Rs. 100,000-250,000 | | -0.844 | -0.181 |
|  |  | (1.134) | (0.468) |
| Between Rs. 250,000-500,000 | | -0.752 | -0.251 |
|  |  | (1.277) | (0.527) |
| More than Rs. 500,000 |  | -0.043 | -0.203 |
|  |  | (1.224) | (0.505) |
| Amount of agricultural land |  | 0.006 | 0.004 |
|  | | (0.020) | (0.008) |
| Total number of poultry |  | 0.023 | 0.016* |
|  | | (0.023) | (0.009) |
| Total number of livestock |  | -0.042 | -0.069* |
|  | | (0.082) | (0.034) |
| Ethnicity (ref: Brahman, Chhetri) | |  |  |
| Newar |  | -0.617 | -0.293 |
|  |  | (0.451) | (0.186) |
| Janajati |  | 0.192 | 0.184 |
|  |  | (0.482) | (0.199) |
| Hill Dalits |  | -1.660 | -0.291 |
|  |  | (1.136) | (0.468) |
| Tarai Dalits |  | -0.563 | -0.134 |
|  |  | -0.512 | -0.211 |
| Mother's education |  | 0.079 | 0.029 |
|  | | (0.052) | (0.021) |
| Household size |  | -0.151* | -0.065* |
|  | | (0.085) | (0.035) |
| Age of the child |  | 0.017 | 0.014 |
|  | | (0.030) | (0.013) |
| Gender of the first respondent | | -0.009 | 0.07 |
|  | | (0.318) | (0.131) |
| Constant | | 4.462*** | 0.532 |
|  |  | (1.337) | (0.552) |
| N |  | 51 | 51 |
| R-squared |  | 0.46 | 0.53 |
|  |  |  |  |
| Note: The table shows results from estimating equation (1) on the sample whose descriptive statistics are in Table (1). Standard errors are in parenthesis. * p<0.10, ** p<0.05, *** p<0.01. Household income and ethnic category are included as categorical variables. In the first column, the coefficient on 'migrant household' should be read as the amount of increase in a child's diet diversity resulting from his or her household member's migration. In the second column, the coefficient multiplied by 100 gives the percentage point change in the probability that the minimum diet diversity is met, again due to a household member's migration. | | | |

**Additional Table S2**. Results from Step-Wise Regressions of Diet Diversity Score on Migration Status

|  | (1) | (2) | (3) | (4) | (5) | (6) | (7) | (8) | (9) | (10) |
| --- | --- | --- | --- | --- | --- | --- | --- | --- | --- | --- |
| Migrant household | 0.734** | 0.733** | 0.750** | 0.775** | 0.885*** | 0.674* | 0.683** | 0.707** | 0.701** | 0.693** |
|  | (0.300) | (0.308) | (0.317) | (0.324) | (0.323) | (0.334) | (0.332) | (0.327) | (0.331) | (0.336) |
| Household income |  | 0.002 | 0.013 | 0.011 | -0.02 | -0.028 | -0.044 | -0.022 | -0.027 | -0.022 |
|  |  | (0.128) | (0.135) | (0.136) | (0.134) | (0.131) | (0.130) | (0.129) | (0.131) | (0.134) |
| Amount of agricultural land | |  | -0.004 | -0.006 | 0.011 | 0.004 | -0.002 | 0.007 | 0.008 | 0.007 |
|  |  |  | (0.015) | (0.016) | (0.018) | (0.018) | (0.018) | (0.019) | (0.020) | (0.020) |
| Total number of poultry |  |  |  | 0.01 | 0.017 | 0.02 | 0.021 | 0.018 | 0.019 | 0.019 |
|  |  |  |  | (0.021) | (0.020) | (0.020) | (0.020) | (0.020) | (0.020) | (0.020) |
| Total number of livestock |  |  |  |  | -0.127* | -0.095 | -0.097 | -0.06 | -0.065 | -0.058 |
|  |  |  |  |  | (0.072) | (0.072) | (0.071) | (0.074) | (0.076) | (0.082) |
| Household's ethnic category | |  |  |  |  | -0.196* | -0.129 | -0.115 | -0.11 | -0.113 |
|  |  |  |  |  |  | (0.106) | (0.117) | (0.115) | (0.117) | (0.119) |
| Mother's education |  |  |  |  |  |  | 0.066 | 0.07 | 0.07 | 0.072 |
|  |  |  |  |  |  |  | (0.050) | (0.049) | (0.050) | (0.050) |
| Household size |  |  |  |  |  |  |  | -0.120 | -0.117 | -0.12 |
|  |  |  |  |  |  |  |  | (0.079) | (0.080) | (0.082) |
| Age of the child |  |  |  |  |  |  |  |  | 0.011 | 0.009 |
|  |  |  |  |  |  |  |  |  | (0.028) | (0.029) |
| Gender of the first respondent | |  |  |  |  |  |  |  |  | 0.079 |
|  |  |  |  |  |  |  |  |  |  | (0.317) |
| Constant | 3.750*** | 3.741*** | 3.721*** | 3.682*** | 3.865*** | 4.523*** | 3.822*** | 4.146*** | 3.956*** | 3.933*** |
|  | (0.234) | (0.605) | (0.615) | (0.626) | (0.620) | (0.701) | (0.875) | (0.888) | (1.012) | (1.028) |
| N | 51 | 51 | 51 | 51 | 51 | 51 | 51 | 51 | 51 | 51 |

| R-squared | 0.11 | 0.11 | 0.11 | 0.12 | 0.17 | 0.23 | 0.26 | 0.30 | 0.30 | 0.30 |
| --- | --- | --- | --- | --- | --- | --- | --- | --- | --- | --- |
| Note: The table shows results from estimating equation (1). Each column represents a separate regression controlling for different set of covariates. Standard errors are in parenthesis. * p<0.10, ** p<0.05, *** p<0.01. Household income and ethnic category are included as continuous values. The coefficient on 'migrant household' should be read as the amount of increase in a child's diet diversity resulting from his or her household member's migration. | | | | | | | | | | |
